# The 2016 CDC Opioid Guideline and Analgesic Prescribing Patterns in Older Adults With Cancer

**DOI:** 10.1001/jamanetworkopen.2025.9043

**Published:** 2025-05-07

**Authors:** Rebecca Rodin, Lihua Li, Karen McKendrick, Krista Harrison, Lauren J. Hunt, Ulrike Muench, Cardinale B. Smith, Melissa D. Aldridge, R. Sean Morrison

**Affiliations:** 1 Brookdale Department of Geriatrics and Palliative Medicine, Icahn School of Medicine at Mount Sinai, New York, New York; 2 Department of Population Health Science and Policy, Icahn School of Medicine at Mount Sinai, New York, New York; 3 Division of Geriatrics, Department of Medicine, University of California, San Francisco; 4 Philip R. Lee Institute for Health Policy Studies, University of California, San Francisco; 5 Department of Epidemiology & Biostatistics, University of California, San Francisco; 6 Division of Hematology and Oncology, Department of Medicine, Icahn School of Medicine at Mount Sinai, New York, New York; 7 Memorial Sloan Kettering Cancer Center, New York, New York; 8 J.J. Peters VA Medical Center, Bronx, New York

## Abstract

**Importance:**

In 2016, the Centers for Disease Control and Prevention (CDC) published guidelines cautioning against prescribing opioids for chronic noncancer pain. Little is known about unintended outcomes of this guideline on analgesic prescribing for older adults with cancer, who commonly require opioids as first-line pain treatment.

**Objective:**

To determine whether the 2016 CDC guideline was associated with altered analgesic prescribing among older adults with cancer.

**Design, Setting, and Participants:**

Interrupted time series analysis of a longitudinal cohort using Medicare Current Beneficiary Survey (MCBS) dataset (2010-2020), a nationally representative longitudinal survey of Medicare beneficiaries linked to Medicare claims. MCBS participants older than 65 years who reported a non–skin cancer diagnosis were followed up for up to 4 years. Subgroup analysis conducted for those with poor prognosis cancer or a cancer-related pain encounter (advanced cancer/cancer pain). Data were analyzed from January 2023 to February 2025.

**Exposure:**

CDC Guideline for Prescribing Opioids for Chronic Pain publication in March 2016.

**Main Outcomes and Measures:**

Quarterly prescribing rates of opioids (typical opioids, tramadol, and buprenorphine) and gabapentinoids (gabapentin and pregabalin). For each time series analysis outcome, a level change estimated immediate change and trend (ie, slope) change estimated ongoing change following the guideline.

**Results:**

The cohort included 11 903 older adults with cancer (mean [IQR] age, 79.4 [73-85] years, 6504 [54.6%] women), including 1283 with advanced cancer or cancer pain. Compared with preguideline trends, we observed the following changes after the guideline release: the slope of opioid prescribing decreased (typical opioids: −0.47; 95% CI, −0.63 to −0.30 percentage points [pp]/quarter; tramadol: −0.27; 95% CI, −0.36 to −0.17 pp/quarter; buprenorphine: −0.01; 95% CI, −0.02 to −0.01 pp/quarter), though tramadol prescribing rose by 11.5% overall; and gabapentinoid prescribing increased by 24.9% (slope change, −0.03; 95% CI, −0.09 to 0.02 pp/quarter).

**Conclusions and Relevance:**

In this cohort study of older adults with cancer, the 2016 CDC guideline was associated with a decline in opioid prescribing that was less pronounced for tramadol compared with typical opioids and was followed by a 25% increase in gabapentinoid prescribing. This may reflect a shift in cancer pain management from first-line opioids to tramadol, which is less safe, and gabapentinoids, which have been shown to be less effective for cancer pain treatment.

## Key Points

**Question:**

Was the 2016 Centers for Disease Control and Prevention (CDC) Guideline for Prescribing Opioids for Chronic Pain associated with unintended analgesic prescribing changes among older adults with cancer?

**Findings:**

This cohort study of 11 903 older adults with cancer using interrupted time series analysis found a significant 24% decline in typical opioid prescribing and in those with advanced disease, an immediate 7.5–percentage-point increase in tramadol prescribing, while gabapentinoid prescribing continued to rise by 25%.

**Meaning:**

These findings suggest the 2016 guideline may have led to pain management shifts from first-line opioids to less-safe tramadol and less-effective gabapentinoids for older adults with cancer.

## Teaser

This cohort study evaluates whether the 2016 US Centers for Disease Control and Prevention (CDC) guideline on prescribing opioids for chronic pain was associated with altered analgesic prescribing among older adults with cancer.

## Introduction

From 1999 to 2021, over half a million people in the US died from an overdose involving a prescription or illicit opioid.^1^ In response, the Centers for Disease Control and Prevention (CDC) published the Guideline for Prescribing Opioids for Chronic Pain in March 2016, recommending cautious use or avoidance of opioids, particularly for adults with chronic noncancer pain. This guideline has been cited as a key factor contributing to a plateauing of prescription opioid-overdose deaths, along with restrictions in state and federal laws, insurance reimbursement of prescription opioids, pharmacy dispensing and monitoring practices, and professional society issuance.^2^

Some research suggests that there may be unintended consequences of the CDC guideline on the legitimate medical use of opioids, including for people with cancer. Studies have shown that the guideline’s recommendations may have been applied to populations for which they were not intended,^3,4^ such as people with sickle cell disease^4^ and those undergoing surgery.^3^ Recent studies have also shown that opioid prescribing has been declining among older adults with cancer, while pain-related emergency department visits in this population have risen.^5,6^ Similarly, from 2013 to 2017, opioid prescriptions by oncologists decreased by 21%, while prescriptions for gabapentin increased by 6%.^7^ Prior studies evaluated the impact of various state and local initiatives, such as prescription drug monitoring programs and Opioid Safety Initiatives, on opioid prescribing in people with cancer, which may partly explain some of these earlier trends.^8,9,10^ However, there are no national data on the subsequent 2016 CDC guideline—one of the few large-scale initiatives with national reach—for the almost 10 million older adults with cancer in the US.^11^

Approximately half of all people with cancer experience pain, with a third reporting moderate-to-severe pain intensity.^12^ Opioids remain the first-line treatment for moderate-to-severe cancer pain^13,14^ and are highly effective, with a reported response rate of 75% and a 50% average reduction in pain intensity.^15,16^ Gabapentinoids, which appear to be increasingly prescribed for cancer pain, are off-label for this indication and have not been shown to demonstrate opioid-sparing analgesic properties except in neuropathic pain.^17,18^ They also have dose-limiting adverse effects, like sedation and confusion, particularly in older adults.^19,20,21^

The CDC clarified that it endorsed recommendations for opioids as first-line therapy for cancer pain in a 2019 response to letters from cancer professional societies and advocacy organizations.^22^ Nevertheless, many opioid restrictions directly based on the CDC guideline remain in place and may inappropriately affect people with cancer. These include state-level restrictions on prescription durations, insurance requirements for prior authorizations, and pharmacies’ more stringent verification processes and opioid dose and duration limits.^23,24,25^

Understanding the specific impact of the 2016 CDC guideline on analgesic prescribing for people with cancer may help to identify the remaining gaps in clinical guideline issuance and support policy initiatives that may help to overcome current barriers to high-quality pain management for this population. We used interrupted time series analysis to examine the association of the 2016 CDC guideline with changes in opioid and gabapentinoid prescribing in a nationally-representative sample of older adults with cancer from 2010 to 2020.

## Methods

### Study Design

This longitudinal cohort study used data from Medicare Current Beneficiary Survey (MCBS), a longitudinal nationally representative survey of Medicare beneficiaries linked to Medicare claims. Survey data are collected from respondents (or proxy) 3 times a year for up to 4 years, with approximately 15 000 participants per year. Prescription drug information is available in the Prescribed Medicine Event file, which integrates both Medicare Part D and survey-reported medications. Self- or proxy-reported medications are verified in person through payment receipts and pill bottles and then further verified by Part D if covered. Of note, MCBS does not include data for the year 2014. The Icahn School of Medicine at Mount Sinai institutional review board approved this study with an informed consent exemption because data were deidentified. This report adhered to the Strengthening the Reporting of Observational Studies in Epidemiology (STROBE) reporting guidelines.^26^

### Sample

We used previously validated methods to identify participants enrolled in MCBS between January 1, 2010, to December 31, 2020, who were aged 65 years or older and reported a cancer diagnosis (excluding skin cancer).^27^ We further identified a subgroup of individuals for whom the benefits of opioids are most likely to outweigh their potential risks: those with poor prognosis cancer or any cancer with a cancer-related pain encounter based on Medicare claims (advanced cancer or cancer pain) (see eAppendix 1 in Supplement 1). We censored participants after death and during any periods of hospice enrollment, as hospice prescribing may have been differentially affected by the CDC guideline.

### Outcomes

We measured the quarterly prescribing rates of opioids (typical opioids [ie, codeine, fentanyl, hydrocodone, hydromorphone, morphine, oxycodone, oxymorphone, or tapentadol], tramadol, and buprenorphine) and gabapentinoids (gabapentin or pregabalin) for up to 1 year after a cancer diagnosis. For buprenorphine, we restricted our analysis to formulations primarily used for pain (see eAppendix 2 in Supplement 1).

We separated tramadol from other opioids because of tramadol’s unique adverse effects (eg, hypoglycemia, serotonin syndrome, and seizures), distinct pharmacodynamics (eg, additional analgesic effect via serotonin and norepinephrine reuptake inhibition), and variable pharmacokinetics (eg, highly polymorphic cytochrome P450 isoenzyme 2D6 metabolism), which makes dosing more difficult than conventional opioids.^28,29,30^ Tramadol also has a distinct regulatory history—it was the only opioid not listed as controlled substance until 2014—that likely contributed to misconceptions about tramadol as a safer opioid.^31,32,33^ These differences may alter tramadol prescribing practices, which may have been differentially affected by the CDC guideline compared with other opioids. Gabapentinoids are adjunct analgesics that are recommended for neuropathic pain syndromes, yet evidence to date has not demonstrated benefit for nonneuropathic pain.^34,35^ See eAppendix 2 in Supplement 1 for full details.

Medications were identified using generic names, with opioid names based on the CDC’s table of commonly prescribed opioids, which account for more than 99% of all opioid prescriptions.^36^ Only prescriptions with complete date information (month, day, and year) were included to ensure accurate quarterly dating. To ensure stability of sample characteristics over time (required for interrupted time series analysis), we collected clinical and sociodemographic characteristics of participants, including age, sex, race and ethnicity, geographic region, and comorbidities. Race and ethnicity were self-reported in MCBS or taken from Medicare administrative data if self-reported data were unavailable and classified as American Indian or Alaskan Native, Asian, Black or African American, Native Hawaiian or Pacific Islander, White, and Other (including multiple races or not otherwise specified).

### Statistical Analysis

We performed quarterly segmented regressions of interrupted time series for each outcome to assess the association between the guideline and prescribing of any opioid, typical opioids, tramadol, buprenorphine, and gabapentinoids. We measured preguideline and postguideline prescribing rates and trends and used the regression coefficients to estimate the magnitude and trajectory of the change in each outcome following the guideline. These include an immediate, 1-time change in mean prescribing rates at the time of the guideline’s publication (ie, level change, or β_2_) and the difference in prescribing trends between the preguideline and postguideline periods (ie, slope change, or β_3_), assuming preguideline trends remained unchanged. We used generalized Durban-Watson statistics to test for potential autocorrelation between successive quarterly measurements. We detected first-order autocorrelation and accounted for it in the analyses. We present unweighted results because our sample included small subgroups for which weighted estimates may not be stable.^37^ Analyses were performed from January 2023 to February 2025 using SAS version 9.4 (SAS Institute).

To evaluate the robustness of our findings, we performed a series of sensitivity analyses. We used an alternate exposure date of December 2015, when a draft of the CDC guideline was announced for public comment. We also used a control outcome because no true control group unexposed to the CDC guideline exists. Although prior studies of the guideline used benzodiazepines as a control outcome,^4,38^ the guideline mentions benzodiazepines at least 35 times, including guidance to avoid their coprescription with opioids.^39^ We therefore chose selective serotonin reuptake inhibitors (SSRIs) because, like opioids, they are psychoactive medications commonly used for symptom management, but do not have analgesic effects^40^ and were not mentioned in the guideline.

## Results

Among the 11 903 people with cancer (mean [IQR] age, 79.4 [73-85] years, 6504 [54.6%] women), there were 1283 people with advanced cancer or cancer pain. Participant characteristics are reported in Table 1 and Table 2.

**Table 1.** Baseline Characteristics of Participants With Reported Cancer Diagnosis by Year

Characteristic	Participants, No. (%) (N = 11 903)									
	2010 (n = 1772)	2011 (n = 1861)	2012 (n = 2046)	2013 (n = 2243)	2015 (n = 2279)	2016 (n = 2084)	2017 (n = 2296)	2018 (n = 2390)	2019 (n = 2586)	2020 (n = 2465)
Any opioid^a^	588 (33.2)	662 (35.6)	750 (36.7)	824 (36.7)	760 (33.4)	678 (32.5)	728 (31.7)	746 (31.2)	706 (27.3)	619 (25.1)
Any typical opioid	498 (28.1)	576 (31.0)	632 (30.9)	689 (30.7)	637 (28.0)	537 (25.8)	585 (25.5)	540 (22.6)	527 (20.4)	462 (18.7)
Any tramadol	142 (8.0)	165 (8.9)	228 (11.1)	242 (10.8)	232 (10.2)	245 (11.8)	240 (10.5)	320 (13.4)	280 (10.8)	234 (9.5)
Any buprenorphine	NA^b^	NA	NA	NA	NA	NA	NA	NA	NA	NA
Any gabapentinoid	155 (8.8)	180 (9.7)	229 (11.2)	258 (11.5)	257 (11.3)	245 (11.8)	289 (12.6)	321 (13.4)	362 (14.0)	350 (14.2)
Any SSRI	255 (14.4)	292 (15.7)	330 (16.1)	347 (15.5)	300 (13.2)	315 (15.1)	347 (15.1)	389 (16.3)	429 (16.6)	377 (15.3)
Age, mean (IQR)	78.7 (72.0-84.0)	78.7 (72.0-84.0)	78.5 (72.0-84.0)	78.3 (71.0-84.0)	78.7 (72.0-84.0)	79.4 (73.0-85.0)	79.6 (73.0-85.0)	79.3 (73.0-85.0)	79.1 (72.0-85.0)	78.9 (70.2-84.0)
Sex										
Female	1019 (57.5)	1033 (55.5)	1119 (54.7)	1250 (55.7)	1222 (53.6)	1117 (53.6)	1288 (56.1)	1314 (55.0)	1416 (54.8)	1344 (54.5)
Male	753 (42.5)	828 (44.5)	927 (45.3)	993 (44.3)	1057 (46.4)	967 (46.4)	1008 (43.9)	1076 (45.0)	1170 (45.2)	1121 (45.5)
Race and ethnicity										
American Indian or Alaskan Native	NA^b^	NA	NA	12 (0.5)	NA	15 (0.7)	12 (0.5)	16 (0.7)	NA	NA
Asian	NA	NA	NA	NA	NA	NA	NA	NA	NA	NA
Black or African American	69 (3.9)	75 (4.0)	84 (4.1)	100 (4.5)	106 (4.7)	104 (5.0)	98 (4.3)	109 (4.6)	113 (4.4)	102 (4.1)
Hispanic	116 (6.6)	121 (6.5)	147 (7.2)	153 (6.8)	134 (5.9)	119 (5.7)	116 (5.1)	126 (5.3)	153 (5.9)	143 (5.8)
Native Hawaiian or Pacific Islander	NA	NA	NA	NA	NA	NA	NA	NA	NA	NA
White	1536 (86.7)	1613 (86.7)	1761 (86.1)	1917 (85.5)	1966 (86.3)	1797 (86.3)	2013 (87.7)	2075 (86.8)	2237 (86.5)	2144 (87.0)
Other^c^	41 (2.3)	40 (2.2)	44 (2.2)	55 (2.5)	61 (2.7)	48 (2.3)	56 (2.4)	64 (2.7)	74 (2.9)	68 (2.8)
Region										
Northeast	276 (15.8)	290 (15.9)	316 (15.7)	360 (16.4)	406 (18.0)	376 (18.2)	418 (18.2)	409 (17.1)	430 (16.6)	445 (18.1)
Midwest	377 (21.6)	438 (23.9)	504 (25.1)	570 (25.9)	569 (25.2)	523 (25.3)	569 (24.8)	583 (24.4)	643 (24.9)	559 (22.7)
South	717 (41.1)	739 (40.4)	780 (38.8)	863 (39.2)	864 (38.3)	772 (37.3)	867 (37.8)	917 (38.4)	997 (38.6)	967 (39.2)
West	374 (21.4)	363 (19.8)	409 (20.4)	409 (18.6)	415 (18.4)	398 (19.2)	442 (19.3)	481 (20.1)	516 (20.0)	494 (20.0)
Poverty^d^	297 (16.8)	291 (15.7)	298 (14.6)	358 (16.0)	475 (20.8)	434 (20.9)	417 (18.2)	387 (16.2)	401 (15.5)	327 (13.3)
Comorbidities										
Diabetes	513 (29.0)	528 (28.4)	636 (31.1)	729 (32.5)	764 (33.5)	701 (34.6)	834 (37.2)	854 (36.6)	939 (36.7)	801 (33.0)
Heart disease	437 (24.7)	449 (24.1)	458 (22.4)	497 (22.2)	813 (35.7)	816 (39.9)	894 (39.7)	859 (36.7)	905 (35.5)	832 (34.3)
Lung disease	368 (20.8)	437 (23.5)	499 (24.4)	530 (23.6)	577 (25.3)	563 (27.8)	630 (28.2)	612 (26.3)	648 (25.4)	596 (24.6)
Stroke	225 (12.7)	257 (13.8)	257 (12.6)	277 (12.4)	297 (13.0)	308 (15.2)	355 (15.9)	339 (14.5)	336 (13.3)	348 (14.4)
Dementia	131 (7.4)	135 (7.3)	121 (5.9)	123 (5.5)	277 (12.2)	137 (6.6)	155 (6.8)	140 (5.9)	127 (4.9)	102 (4.1)
Hospice	35 (2.0)	44 (2.4)	37 (1.8)	47 (2.1)	23 (1.0)	30 (1.4)	32 (1.4)	44 (1.8)	40 (1.6)	41 (1.7)
Deaths	49 (2.8)	52 (2.8)	55 (2.7)	58 (2.6)	33 (1.5)	45 (2.2)	41 (1.8)	41 (1.7)	44 (1.7)	49 (2.0)
Inpatient	360 (20.3)	388 (20.9)	388 (19.0)	467 (20.8)	427 (18.7)	361 (17.3)	384 (16.7)	427 (17.9)	440 (17.0)	326 (13.2)
SNF	116 (6.6)	128 (6.9)	140 (6.8)	150 (6.7)	148 (6.5)	114 (5.5)	157 (6.8)	154 (6.4)	121 (4.7)	93 (3.8)

Abbreviations: NA, not available; SNF, skilled nursing facility; SSRI, selective serotonin reuptake inhibitor.

^a^ Any opioid refers to any prescription for a typical opioid (codeine, fentanyl, hydrocodone, hydromorphone, morphine, oxycodone, oxymorphone, or tapentadol), tramadol, or buprenorphine (transdermal patch or buccal film).

^b^ Small cell sizes (n < 11) are suppressed in accordance with the Centers for Medicare and Medicaid Services policy.

^c^ Other included those with multiple or not otherwise specified race(s) or ethnicity(s).

^d^ Poverty was defined as an annual income less than $15 000 for unmarried individuals and less than $20 000 for married individuals based on approximations of the federal poverty level.

**Table 2.** Baseline Characteristics of Participants With Advanced Cancer or Cancer Pain (n = 1283) by Year

Characteristic	Participants, No. (%) (N = 1283)									
	2010 (n = 142)	2011 (n = 202)	2012 (n = 208)	2013 (n = 248)	2015 n = 217)	2016 (n = 203)	2017 (n = 193)	2018 (n = 195)	2019 (n = 192)	2020 (n = 182)
Any opioid^a^	69 (48.6)	97 (48.0)	103 (49.5)	132 (53.2)	91 (41.9)	96 (47.3)	87 (45.1)	94 (48.2)	70 (36.5)	62 (34.1)
Any typical opioid	63 (44.4)	92 (45.5)	94 (45.2)	119 (48.0)	79 (36.4)	78 (38.4)	71 (36.8)	70 (35.9)	53 (27.6)	55 (30.2)
Any tramadol	14 (9.9)	21 (10.4)	24 (12.0)	32 (12.9)	19 (8.8)	36 (17.7)	25 (13.0)	42 (23.2)	29 (15.1)	18 (9.9)
Any buprenorphine	NA^b^	NA	NA	NA	NA	NA	NA	NA	NA	NA
Any gabapentinoid	15 (10.6)	23 (11.4)	34 (16.4)	37 (14.9)	34 (15.7)	28 (13.8)	30 (15.5)	31 (15.9)	34 (17.7)	29 (15.9)
Any SSRI	17 (12.0)	31 (15.4)	35 (16.8)	46 (18.6)	29 (13.4)	39 (19.2)	34 (17.6)	37 (19.0)	32 (16.7)	31 (17.0)
Age, mean (IQR)	79.7 (74.0-85.0)	78.7 (72.0-83.0)	78.8 (72.0-84.0)	78.4 (72.0-84.0)	78.5 (72.0-84.0)	79.6 (74.0-85.0)	81.1 (76.0-86.0)	80.2 (74.0-85.0)	79.6 (73.0-86.0)	79.7 (73.0-85.0)
Sex										
Female	75 (52.8)	112 (55.5)	119 (57.2)	140 (56.5)	122 (56.2)	117 (57.6)	113 (58.6)	103 (52.8)	108 (56.3)	109 (59.9)
Male	67 (47.2)	90 (44.6)	89 (42.8)	108 (43.8)	95 (43.8)	86 (42.4)	80 (41.5)	92 (47.2)	84 (43.8)	73 (40.1)
Race and ethnicity										
American Indian or Alaskan Native	NA^b^	NA	NA	NA	NA	NA	NA	NA	NA	NA
Asian	NA	NA	NA	NA	NA	NA	NA	NA	NA	NA
Black or African American	15 (10.6)	20 (9.9)	13 (6.3)	23 (9.3)	17 (7.8)	13 (6.4)	17 (8.8)	15 (7.7)	19 (9.9)	13 (7.1)
Hispanic	NA	17 (8.4)	21 (10.1)	23 (9.3)	20 (9.2)	21 (10.3)	20 (10.4)	22 (11.3)	15 (7.8)	13 (7.1)
Native Hawaiian or Pacific Islander	NA	NA	NA	NA	NA	NA	NA	NA	NA	NA
White	112 (78.9)	157 (77.7)	168 (80.8)	192 (77.4)	170 (78.3)	158 (77.8)	148 (76.7)	151 (77.4)	150 (78.1)	149 (81.9)
Other^c^	NA	NA	NA	NA	NA	NA	NA	NA	NA	NA
Region										
Northeast	27 (19.2)	38 (18.8)	38 (18.4)	51 (20.7)	47 (21.8)	41 (20.2)	35 (18.1)	44 (22.6)	52 (27.1)	45 (24.7)
Midwest	38 (27.0)	57 (28.2)	57 (27.5)	76 (30.9)	54 (25.0)	54 (26.6)	51 (26.4)	45 (23.1)	43 (22.4)	45 (24.7)
South	51 (36.2)	73 (36.1)	81 (39.1)	88 (35.8)	83 (38.4)	70 (34.5)	77 (39.9)	70 (35.9)	69 (35.9)	63 (34.6)
West	25 (17.7)	34 (16.8)	31 (15.0)	31 (12.6)	32 (14.8)	38 (18.7)	30 (15.5)	36 (18.5)	28 (14.6)	29 (15.9)
Poverty^d^	40 (28.2)	43 (21.4)	42 (20.2)	47 (19.0)	76 (35.0)	52 (25.7)	53 (27.6)	44 (22.7)	45 (23.6)	36 (19.9)
Comorbidities										
Diabetes	36 (25.4)	59 (29.4)	74 (35.6)	90 (36.3)	74 (34.1)	73 (39.9)	83 (45.6)	77 (42.3)	73 (40.8)	58 (34.1)
Heart disease	57 (40.1)	60 (29.7)	63 (30.3)	74 (29.8)	105 (48.6)	115 (60.5)	112 (60.2)	105 (56.8)	105 (58.3)	104 (59.8)
Lung disease	33 (23.2)	62 (30.9)	76 (36.5)	95 (38.3)	86 (39.8)	83 (44.6)	76 (42.5)	84 (45.4)	80 (44.4)	71 (41.8)
Stroke	17 (12.0)	25 (12.4)	28 (13.5)	27 (10.9)	33 (15.3)	26 (14.4)	37 (19.9)	30 (16.5)	29 (16.8)	31 (18.9)
Dementia	13 (9.2)	15 (7.4)	20 (9.6)	24 (9.7)	38 (17.5)	15 (7.4)	19 (9.8)	13 (6.7)	14(7.3)	NA
Hospice	16 (11.3)	21 (10.4)	16 (7.7)	31 (12.5)	NA	14 (6.9)	14 (7.3)	18 (9.2)	16 (8.3)	15 (8.2)
Deaths	15 (10.6)	17 (8.4)	12 (5.8)	24 (9.7)	NA	19 (9.4)	15 (7.8)	16 (8.2)	NA	14 (7.7)
Inpatient	68 (47.9)	85 (42.1)	86 (41.4)	116 (46.8)	80 (36.9)	86 (42.4)	63 (32.6)	60 (30.8)	63 (32.8)	55 (30.2)
SNF	66 (46.5)	82 (40.6)	86 (41.4)	112 (45.2)	73 (33.6)	81 (39.9)	58 (30.1)	57 (29.2)	60 (31.3)	50 (27.5)

Abbreviations: NA, not available; SNF, skilled nursing facility; SSRI, selective serotonin reuptake inhibitor.

^a^ Any opioid refers to any prescription for a typical opioid (codeine, fentanyl, hydrocodone, hydromorphone, morphine, oxycodone, oxymorphone, or tapentadol), tramadol, or buprenorphine (transdermal patch or buccal film).

^b^ Small cell sizes (n <11) are suppressed in accordance with the Centers for Medicare and Medicaid Services policy.

^c^ Other included those with multiple or not otherwise specified race(s) or ethnicity(s).

^d^ Poverty was defined as an annual income less than $15 000 for unmarried individuals and less than $20 000 for married individuals based on approximations of the federal poverty level.

### Analgesic Prescribing Patterns Before and After the CDC Guideline

The Figure and eFigure in Supplement 1 show the proportions of people prescribed opioids or gabapentinoids from 2010 to 2020 for older adults with any cancer and advanced cancer or cancer pain, respectively. From the preguideline to postguideline period, the mean proportion of older adults prescribed a typical opioid decreased from 29.4% (95% CI, 28.7%-30.1%) to 22.4% (95% CI, 21.0%-23.8%) for any cancer and 42.8% (95% CI, 40.8%-44.8%) to 32.7% (95% CI, 30.3%-35.1%) for advanced cancer or cancer pain, representing 23.8% and 23.5% drops, respectively (Table 3). Mean prescribing rates for tramadol increased from 10.0% (95% CI, 9.4%-10.6%) to 11.1% (95% CI, 10.5%-11.8%) for any cancer and 11.3% (95% CI, 10.4%-12.2%) to 15.9% (95% CI, 13.4%-18.3%) for advanced cancer or cancer pain. Mean buprenorphine prescribing remained near 0 during the study period. By contrast, gabapentinoid prescribing rates increased by 24.9% and 12.2% from the preguideline to postguideline periods for those with any cancer and advanced cancer or cancer pain, respectively (Table 3).

**Figure.**
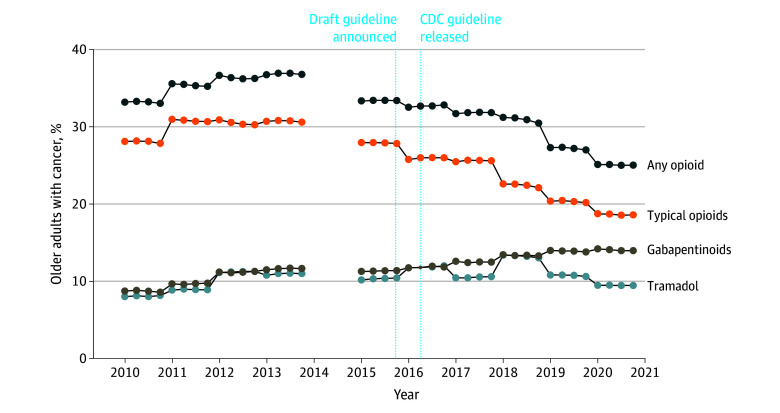
Analgesic Prescribing in Older Adults With Any Cancer, 2010 to 2020 The Medicare Current Beneficiary Survey does not include data for 2014. All percentages are descriptive (ie, not modeled). Any opioid includes typical opioids, tramadol, and buprenorphine. Buprenorphine is not individually depicted because its prescribing rate remained near 0 throughout the study period (see Tables 1, 2, and 3). CDC indicates US Centers for Disease Control and Prevention.

**Table 3.** Interrupted Time Series Analysis of Opioid and Gabapentinoid Prescribing Before and After the 2016 US Centers for Disease Control and Prevention Opioid Guideline

Analgesic prescription	Preguideline mean, constant (95% CI), %^c^	Preguideline trajectory, β_1_^a^		Postguideline mean, constant (95% CI)^e^	Postguideline trajectory, β_1 + 3_^b^		Change associated with guideline release				
		Slope (95% CI)^d^	*P* value		Slope (95% CI)^d^	*P* value	Immediate level change^f^ (95% CI)	*P* value	Change in slope, β_3_ (95% CI)	*P* value	Relative level change, %^g^
**Patients with any cancer (n = 11 903)**											
Any opioid^h^	34.9 (34.2 to 35.6)	0.04 (−0.12 to 0.20)	.61	29.4 (27.9 to 30.8)	−0.48 (−0.56 to −0.40)	<.001	−0.87 (−3.00 to 1.25)	.43	−0.52 (−0.71 to −0.34)	<.001	−15.8
Typical opioid	29.4 (28.7 to 30.1)	−0.01 (−0.16 to 0.14)	.88	22.4 (21.0 to 23.8)	−0.48 (−0.54 to −0.41)	<.001	−1.87 (−3.64 to −0.10)	.05	−0.47 (−0.63 to −0.30)	<.001	−23.8
Tramadol	10.0 (9.4 to 10.6)	0.16 (0.09 to 0.23)	<.001	11.1 (10.5 to 11.8)	−0.11 (−0.18 to −0.04)	<.001	0.90 (−0.35 to 2.15)	.17	−0.27 (−0.36 to −0.17)	<.001	11.5
Buprenorphine	0.06 (0.01 to 0.11)	0.01 (0.01 to 0.02)	.001	0.05 (0.03 to 0.07)	0.00 (0.00 to 0.00)	.92	−0.14 (−0.25 to −0.03)	.02	−0.01 (−0.02 to −0.01)	.003	−19.0
Gabapentinoid	10.6 (10.0 to 11.1)	0.18 (0.13 to 0.22)	<.001	13.2 (12.8 to 13.6)	0.14 (0.12 to 0.16)	<.001	−0.49 (−1.13 to 0.14)	.14	−0.03 (−0.09 to −0.02)	.23	24.9
SSRIs	14.8 (14.4 to 15.3)	−0.07 (−0.16 to 0.03)	.18	15.6 (15.3 to 15.9)	0.02 (−0.04 to 0.09)	.47	1.13 (−0.05 to 2.30)	.07	0.09 (−0.02 to 0.21)	.14	5.0
**Patients with advanced cancer or cancer pain (n = 1283)**											
Any Opioid	47.5 (45.6 to 49.3)	−0.05 (−0.42 to 0.32)	.80	41.0 (38.0 to 44.0)	−0.90 (−1.10 to −0.71)	<.001	3.82 (−2.16 to 9.79)	.22	−0.85 (−1.29 to −0.42)	<.001	−13.6
Typical opioid	42.8 (40.8 to 44.8)	−0.22 (−0.62 to 0.18)	.29	32.7 (30.3 to 35.1)	−0.74 (−0.96 to −0.53)	<.001	−0.12 (−5.83 to 5.60)	.97	−0.52 (−0.98 to −0.06)	.04	−23.5
Tramadol	11.3 (10.4 to 12.2)	0.07 (−0.06 to 0.20)	.32	15.9 (13.4 to 18.3)	−0.31 (−0.57 to −0.04)	.03	7.51 (3.46 to 11.56)	.001	−0.37 (−0.67 to −0.08)	.02	40.0
Buprenorphine	0.00 (0.00 to 0.00)	0.00 (0.00 to 0.00)	NA	0.00 (0.00 to 0.00)	0.00 (0.00 to 0.00)	NA	0.00 (0.00 to 0.00)	NA	0.00 (0.00 to 0.00)	NA	0.0
Gabapentinoid	14.3 (13.0 to 15.6)	0.37 (0.29 to 0.46)	<.001	16.1 (15.6 to 16.5)	0.12 (0.04 to −0.21)	.004	−3.26 (−4.63 to −1.90)	<.001	−0.25 (−0.37 to −0.13)	<.001	12.2
SSRIs	15.1 (13.9 to 16.2)	0.15 (−0.08 to 0.38)	.22	18.0 (17.2 to 18.8)	−0.22 (−0.29 to −0.15)	<.001	4.08 (1.27 to 6.90)	.007	−0.37 (−0.62 to −0.13)	.005	19.3

Abbreviations: NA, not applicable; SSRIs, selective serotonin reuptake inhibitors.

^a^ The preguideline period was from January 2010 to March 2016 for all outcomes.

^b^ The postguideline period was from April 2016 to December 2020 for all outcomes.

^c^ Preguideline mean is the mean prescribing rate in the preguideline period.

^d^ Slopes represent the change in the indicated variable per quarter (ie, 3 months).

^e^ The postguideline mean is the mean prescribing rate in the postguideline period.

^f^ Immediate level change (ie, β_2_) represents a one-time change at the time of the guideline’s release (ie, March 2016).

^g^ Relative change represents the change in prescribing rate in the post- compared with preguideline period.

^h^ Any opioid includes typical opioids (codeine, fentanyl, hydrocodone, hydromorphone, morphine, oxycodone, oxymorphone, or tapentadol), tramadol, and buprenorphine (transdermal patch or buccal film).

### Changes in Opioid Prescribing Associated With the CDC Guideline

Among those with any cancer, the 2016 guideline was associated with a subsequent decrease in trend (ie, slope) for prescribing of any opioid (−0.52; 95% CI, −0.71 to −0.34 percentage points [pp]/quarter; *P* < .001), including typical opioids (−0.47; 95% CI, −0.63 to −0.30 pp/quarter; *P* < .001), tramadol (−0.27; 95% CI, −0.36 to −0.17 pp/quarter; *P* < .001), and buprenorphine (−0.01; 95% CI, −0.02 to −0.01 pp/quarter; *P* = .003). Similar findings were observed in those with advanced cancer or cancer pain, but with a steeper decline in any opioid prescribing (−0.85; 95% CI, −1.29 to −0.42 pp/quarter; *P* < .001) and an immediate increase in tramadol prescribing postguideline (level change, 7.51; 95% CI, 3.46-11.56 pp; *P* = .001) (Table 3).

### Changes in Gabapentinoid Prescribing Associated With the CDC Guideline

Gabapentinoid prescribing in those with any cancer continued to increase following the guideline, but without a significant change in slope between the preguideline and postguideline periods (slope change, −0.03; 95% CI, −0.09 to −0.02; pp/quarter; *P* = .23) (Table 3). In those with advanced cancer or cancer pain, this growth continued at a slower rate in the postguideline period (slope change, −0.25; 95% CI, −0.37 to −0.13 pp/quarter; *P* < .001) (Table 3). One-time level changes associated with the guideline are reported in Table 3.

### Sensitivity Analyses

Results from the sensitivity analyses were similar to those reported in the main analyses, except that there was no change in tramadol prescribing associated with the guideline among those with advanced cancer or cancer pain (slope change, −0.22; 95% CI, −0.62 to 0.18 pp/quarter; *P* = .28; level change, 4.45; 95% CI, −0.28 to 9.17 pp; *P* = .08) (eTable in Supplement 1). For SSRI prescribing, there was no change associated with the CDC guideline in those with any cancer (slope change, 0.10; 95% CI, −0.05 to 0.24 pp/quarter; *P* = .21; level change, −0.29; 95% CI, −1.69 to 1.12 pp; *P* = .70) and advanced cancer or cancer pain (slope change, −0.32; 95% CI, −0.68 to 0.03 pp/quarter; *P* = .09; level change, 1.10; 95% CI, −2.75 to 4.96 pp; *P* = .58) (Table 3). Results for SSRI prescribing using the alternate exposure date are reported in the eTable in Supplement 1.

## Discussion

This cohort study is the first study we know of with a nationally representative population to show that the CDC guideline release was associated with a reduction in opioid prescribing in older adults with cancer. We found that the publication of the 2016 CDC guideline was associated with an overall decrease in opioid prescribing among older adults with cancer, including those with advanced cancer or documented cancer pain. However, tramadol prescribing was relatively preserved overall, even increasing in the immediate postguideline period in those with advanced cancer or cancer pain. At the same time, gabapentinoid prescribing rose by 25% and 12% among those with any cancer and advanced cancer or cancer pain, respectively.

Possible mechanisms by which opioid prescribing may have decreased following the CDC guideline include a direct effect of the guideline on physicians’ prescribing behaviors and indirect effects on subsequent opioid-related initiatives. The latter include pharmacies’ opioid stocking and dispensing changes following multibillion dollar settlements in 2017 through 2022,^41^ the Drug Enforcement Agency (DEA)’s reduction in opioid manufacturing quotas since 2017,^42,43,44,45^ ongoing litigation against opioid manufacturers,^46^ state legislation enacting opioid excise taxes or fees in 2019 through 2020,^47^ and the publication of best-selling books on this subject.^48,49^ In many cases, these initiatives were based directly on the 2016 CDC guideline, such as the DEA’s decision to reduce opioid manufacturing,^42,43,44,45^ state limits on opioid doses and durations,^50^ and changes to pharmacy dispensing practices.^23^ Indeed, pain experts, task forces, consensus panels, and the CDC itself have all suggested that the 2016 guideline was broadly adopted by health insurance companies, state legislatures, health systems, and clinicians and inappropriately applied to people with cancer.^2,25,50,51^

The ongoing growth of gabapentinoid prescribing from 2010 to 2020 in this study mirrors its rise in off-label use in people with cancer reported elsewhere.^34,52^ Initially approved for epilepsy and then for postherpetic neuralgia and diabetic neuropathy, gabapentin became the tenth most prescribed medication in the US and brand-name pregabalin (Lyrica) was eighth in drug spending in 2016.^35^ However, gabapentinoid use is off-label for cancer pain with limited evidence for its analgesic efficacy.^34,53,54^ Indeed, the few studies on gabapentinoids for treating neuropathic cancer pain show analgesic benefit mainly in combination with opioid therapy,^54,55,56^ or have small sample sizes with limited opioid-sparing analgesic effect (eg, <1 point of 10 difference in average pain intensity compared with placebo).^57^ By contrast, opioids reduce average pain intensity by 50%, or 3 points of 10 among people with cancer.^15,58^ Gabapentinoids also carry important risks of sedation, dizziness, and confusion, particularly among older adults.^19,20,21^

The ongoing growth of gabapentinoids in the context of declining opioid prescribing raises concern that less-effective gabapentinoid therapies may have been substituted for first-line opioids for cancer pain. This may be because gabapentinoids faced less regulatory scrutiny than opioids and have fewer prior authorization requirements. Health care clinicians may also have sought to avoid opioid-related litigation by more closely aligning with what they believed to be the CDC guideline recommendations.

The more modest decline in tramadol prescribing compared with that of typical opioids may indicate preferential selection of tramadol over safer, more predictably effective opioids. This is further supported by the 1-time increase in tramadol prescribing observed immediately after the guideline in those with advanced cancer or cancer pain, despite declining trends for typical opioids. This is concerning as tramadol has weaker analgesic properties than typical opioids, an equal risk for abuse, more variable pharmacokinetics, and unique risks of serotonin syndrome, hyponatremia, seizures, and hypoglycemia—particularly in older adults.^28,29,30^

Preferential selection of tramadol over other opioids may be due to differences in their regulatory oversight and scrutiny. Tramadol was an uncontrolled substance until 2014, when the DEA classified it as a Schedule IV drug, unlike other opioids that have long been classified as Schedule II.^33,59^ This regulatory difference was based on a misperception of tramadol as a weak and therefore safer opioid with a low risk for addiction, which was how it was marketed by its manufacturers, some of whom are now facing lawsuits for allegedly misleading campaigns.^31,60^ This perception of tramadol may persist among clinicians who may incorrectly view tramadol as a safer opioid, despite its unique risks.

In November 2022, the CDC published a revised opioid prescribing guideline in response to concerns about unintended harms from its 2016 guideline. This revised guideline acknowledges, based on expert consensus, that the 2016 guideline was inappropriately applied to people with cancer.^36^ Yet, it does not provide explicit corrective recommendations, other than to link to professional society guidelines in oncology,^18,61,62,63^ which do recommend opioid therapy for moderate to severe cancer pain. More concerning is that neither the 2022 CDC guideline nor the linked oncology guidelines provide clear guidance regarding the use of tramadol over other opioids or discuss tramadol’s potentially life-threatening complications.^18^ These guidelines also do not discuss gabapentinoids’ adverse effects, particularly for older adults. Such guidance may help to improve analgesic prescribing practices for all people with cancer.

### Strengths and Limitations

Strengths of this study are the inclusion of a nationally representative sample of older patients with cancer and the design using an interrupted time series approach. Due to the observational nature of this study, we were unable to determine causation between the CDC guideline and analgesic prescribing outcomes. However, our interrupted time series approach strengthens the study by detecting trend shifts associated with clinical events.^64^ Other opioid-related initiatives not affected by the CDC guideline may have confounded our findings. This potential for confounding is an inherent feature in many health policy analyses, including those related to opioid prescribing, when no control population exists and randomized controlled trials are not possible.^65,66,67^ We therefore attempted to strengthen our study with sensitivity analyses using an alternate exposure date and a control outcome, which supported our main findings.^4,38^

Other limitations include that MCBS lacks data on cancer stage, initial date of cancer diagnosis, symptom burden, and prescription indication, which may affect the interpretation of prescribing outcomes. We also lacked data on pain burden and intensity, and thus could not assess the impact of prescribing changes on these outcomes.

We did not measure all buprenorphine formulations or methadone, which may have increased during this period due to the inability of determining their indication for either substance use disorder or pain. However, the relative impact of these medications would be expected to be small because of additional regulatory and training requirements needed to prescribe them during this time.^68^ Similarly, we did not measure corticosteroid, tricyclic antidepressant (TCA), or serotonin norepinephrine reuptake inhibitor (SNRI) prescribing due to the inability of determining their primary indication (eg, depression, pain, or inflammatory disease). These medications are also unlikely to have significantly affected our results because of their relatively narrow therapeutic indications (eg, SNRIs for neuropathic pain or corticosteroids for short-term use in inflammatory pain)^69,70^ and frequent contraindications in older adults (eg, TCAs).^71^ Additionally, analgesic efficacy of antidepressants for chronic pain has been demonstrated only for SNRIs, not TCAs.^40^ Finally, we did not account for acetaminophen or nonsteroidal anti-inflammatory analgesics, which are typically purchased over-the-counter, as these medications have relatively little efficacy for cancer pain treatment in older adults.^72,73,74^

## Conclusions

In this cohort study of older adults with cancer, the 2016 CDC guideline was associated with a decline in first-line opioids while less-safe tramadol and less-effective gabapentinoid prescribing continued to rise. Further revision of the recent 2022 CDC guideline and oncology pain management guidelines may be needed to help address these potentially inappropriate analgesic shifts.

## Notes

Supplement 1. **eAppendix 1. **Additional detail on sample selection **eAppendix 2. **Additional detail on prescribing outcomes **eFigure.** Analgesic prescribing in older adults with advanced cancer or cancer pain, 2010 to 2020 **eTable. **Interrupted time series analysis of opioid and anticonvulsant prescribing in older adults with cancer and advanced cancer or cancer pain before and after the December 2015 CDC guideline draft release
eReferences

Supplement 2.
Data Sharing Statement
