# Supplementary material for: The 2016 CDC Opioid Guideline and Analgesic Prescribing Patterns in Older Adults With Cancer
Source: JAMA Netw Open. 2025 May 7;8(5):e259043. doi: 10.1001/jamanetworkopen.2025.9043 (PMC12059969; doi:10.1001/jamanetworkopen.2025.9043)
Supplement: Supplement 1. — eAppendix 1. Additional detail on sample selection eAppendix 2. Additional detail on prescribing outcomes eFigure. Analgesic prescribing in older adults with advanced cancer or cancer pain, 2010 to 2020 eTable. Interrupted time series analysis of opioid and anticonvulsant prescribing in older adults with cancer and advanced cancer or cancer pain before and after the December 2015 CDC guideline draft release eReferences [file jamanetwopen-e259043-s001.pdf]

Supplemental Online Content

Rodin R, Li L, McKendrick K, et al. The 2016 CDC opioid guideline and analgesic prescribing patterns in older adults with cancer. *JAMA Netw Open*. 2025;8(5):e259043. doi:10.1001/jamanetworkopen.2025.9043

eAppendix 1. Additional detail on sample selection
eAppendix 2. Additional detail on prescribing outcomes
eFigure. Analgesic Prescribing in Older Adults with Advanced Cancer or Cancer Pain, 2010 to 2020
eTable. Interrupted Time Series Analysis of Opioid and Anticonvulsant Prescribing in Older Adults with Cancer and Advanced Cancer or Cancer Pain Before and After the December 2015 CDC Guideline Draft Release
eReferences

This supplemental material has been provided by the authors to give readers additional information about their work.

© 2025 Rodin R et al. *JAMA Network Open*.

eAppendix 1. Additional detail on sample selection

As in prior studies, we used International Classification of Disease Ninth (ICD-9) or Tenth (ICD-10) Revision codes to identify adults ≥65 years old who were continuously enrolled in Medicare Part D and who had ≥1 inpatient or ≥2 outpatient claims for poor-prognosis cancer.[1-3] A similar approach, using ICD-9 and ICD-10 codes adapted from SEER-Medicare,[4] was used to identify individuals with any malignancy with ≥1 inpatient or outpatient claim for cancer-related pain (ICD-9 338.3, ICD-10 G89.3). We removed all diagnosis codes relating to a 'personal history of' cancer in order to minimize the inclusion of participants whose cancer was cured or in remission, as such individuals are less likely to have moderate-to-severe cancer-related pain requiring opioid therapy. For participants with hospice enrollment or death occurring mid-quarter, we censored their data if more than half of the days in that quarter (i.e., ≥46 days) were spent enrolled in hospice or after date of death.

© 2025 Rodin R et al. *JAMA Network Open*.

eAppendix 2. Additional detail on prescribing outcomes

Due to the difficulty of determining whether buprenorphine medications were prescribed for pain or substance use disorder treatment, we restricted our analysis to the low-dose formulations of buprenorphine that are primarily used for analgesia – buprenorphine transdermal patch (e.g., Butrans)] and buccal film (e.g., Belbuca). We similarly excluded methadone because of the inability to determine whether it was used for pain or substance use disorder without access to the medical record.

We used anticonvulsants gabapentin and pregabalin as a measure of non-opioid analgesic prescribing, as these are the ones used for the management of cancer pain.[5] We did not measure prescribing of adjuvant corticosteroids, tricyclic antidepressants (e.g., amitriptyline), and serotonin norepinephrine reuptake inhibitors (e.g., duloxetine) due to an inability of determining their primary indication (e.g., depression or pain).

© 2025 Rodin R et al. *JAMA Network Open*.

eFigure 1. Analgesic Prescribing in Older Adults with Advanced Cancer or Cancer Pain, 2010 to 2020

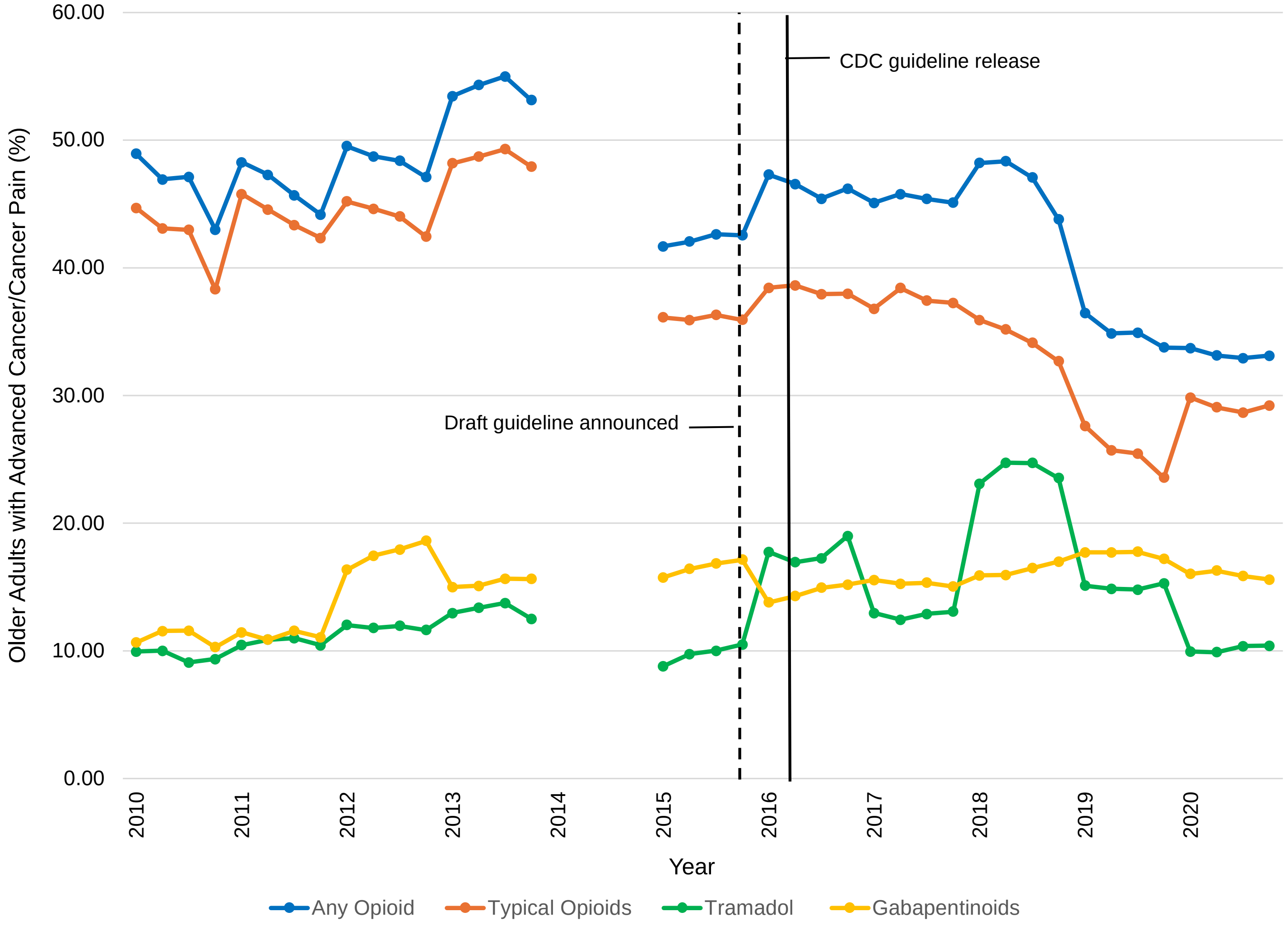

* All percentages are descriptive (i.e., not modeled)
**Any opioid includes typical opioids, tramadol, and buprenorphine
***Buprenorphine is not individually depicted because its prescribing rate remained near zero throughout the study period (see Tables 1-3)
****Data for the year 2014 are not available in Medicare Current Beneficiary Survey

© 2025 Rodin R et al. *JAMA Network Open*.

eTable 1. Interrupted Time Series Analysis of Opioid and Anticonvulsant Prescribing in Older Adults with Cancer and Advanced Cancer or Cancer Pain Before and After the December 2015 CDC Guideline Draft Release

Analgesic Prescribing Rate, %	Pre-Guideline Mean[a]	Preguideline Trajectory[b]		Post-Guideline Mean[c]	Postguideline Trajectory[d]		Change Associated with Guideline Release				
	Constant (95% CI)	Slope[e] (95% CI)	*P*	Constant (95% CI)	Slope[e] (95% CI)	*P*	Level change[f] (95% CI)	*P*	Change in slope (95% CI)	*P*	Relative Level Change[g]
**Any Cancer**											
Any Opioid	35.1 (34.4-35.9)	0.13 (-0.06 to 0.31)	0.19	29.7 (28.3-31.1)	-0.45 (-0.52 to -0.38	<.001	-1.25 (-3.39 to 0.89	0.26	-0.57 (-0.78 to -0.37)	<.001	-15.4%
Typical opioid	29.7 (29.1-30.3)	0.05 (-0.11 to 0.22)	0.53	22.8 (21.4-24.2)	-0.48 (-0.52 to -0.44)	<.001	-1.68 (-3.45 to 0.10)	0.08	-0.53 (-0.71 to -0.36)	<.001	-23.1%
Tramadol	9.9 (9.3-10.5)	0.20 (0.12-0.27)	<.001	11.1 (10.5-11.7)	-0.06 (-0.14 to -0.01)	0.12	0.31 (-0.99 to 1.62)	0.64	-0.26 (-0.38 to -0.15)	<.001	12.8%
Buprenorphine	0.05 (0.00-0.10)	0.01 (0.00-0.02)	0.04	0.06 (0.04 to 0.09)	0.00 (-0.01 to 0.00)	0.16	0.01 (-0.13 to 0.14)	0.94	-0.01 (-0.03 to 0.00)	0.02	28.0%
Gabapentinoid	10.5 (9.9-11.0)	0.20 (0.15-0.25)	<.001	13.1 (12.6-13.5)	0.14 (0.12-0.17)	<.001	-0.82 (-1.43 to -0.22)	0.02	-0.06 (-0.00 to 0.00)	0.05	24.7%
SSRIs	14.9 (14.5-15.4)	-0.02 (-0.14 to 0.09)	0.67	15.5 (15.1-15.8)	0.07 (-0.01 to 0.16)	0.10	-0.29 (-1.69 to 1.12)	0.70	0.10 (-0.05 to 0.24)	0.21	3.6%
**Advanced Cancer/Cancer Pain**											
Any Opioid	47.7 (45.8-49.7)	0.13 (-0.35 to 0.62)	0.60	41.4 (38.7-44.2)	-0.71 (-0.98 to -0.43)	<.001	0.41 (-6.28 to 7.09)	0.91	-0.84 (-1.43 to -0.25)	0.008	-13.3%
Typical opioid	43.4 (41.4-45.3)	-0.02 (-0.51 to 0.48)	0.95	33.1 (30.9-35.4)	-0.63 (-0.84 to -0.41)	<.001	-3.10 (-9.27 to 3.07)	0.34	-0.61 (-1.17 to -0.05)	0.04	-23.6%
Tramadol	11.0 (10.3-11.7)	0.12 (-0.05 to 0.30)	0.19	15.7 (13.4-17.9)	-0.10 (-0.44 to 0.24)	0.57	4.45 (-0.28 to 9.17)	0.08	-0.22 (-0.62 to 0.18)	0.28	42.2%
Buprenorphine	0.00 (0.0)	0.00 (0.00-0.00)	N/A	0.00 (0.0)	0.00 (0.00-0.00)	N/A	0.00 (0.00-0.00)	N/A	0.00 (0.00-0.00)	N/A	0.0%
Gabapentinoid	14.2 (12.8-15.6)	0.40 (0.29-0.52)	<.001	16.0 (15.5-16.5)	0.07 (-0.02 to 0.16)	0.14	-2.25 (-4.36 to -0.14)	0.05	-0.33 (-0.48 to -0.19)	<.001	12.7%
SSRIs	14.9 (13.7-16.1)	0.26 (0.00-0.52)	0.06	17.9 (17.0-18.7)	-0.06 (-0.27 to 0.15)	0.56	1.10 (-2.75 to 4.96)	0.58	-0.32 (-0.68 to 0.03	0.09	19.6%

[a]The pre-guideline mean is the mean prescribing rate in the pre-guideline period
[b]The pre-guideline period was from January 2010 to December 2015 for all outcomes
[c]The post-guideline mean is the mean prescribing rate in the post-guideline period
[d]The post-guideline period was from January 2016 to December 2020 for all outcomes
[e]Slopes represent the change in the indicated variable per quarter (i.e., 3 months)
[f]Immediate level change represents a one-time change at the time of the guideline's release (i.e., March 2016)
[g]Relative change represents the change in prescribing rate in the post- compared to pre-guideline period
SSRI = selective serotonin reuptake inhibitors; N/A = not applicable

© 2025 Rodin R et al. *JAMA Network Open*.

eReferences

1. Enzinger AC, Ghosh K, Keating NL, Cutler DM, Landrum MB, Wright AA. US Trends in Opioid Access Among Patients With Poor Prognosis Cancer Near the End-of-Life. *J Clin Oncol*. Sep 10 2021;39(26):2948-2958. doi:10.1200/jco.21.00476
2. Enzinger AC, Ghosh K, Keating NL, et al. Racial and Ethnic Disparities in Opioid Access and Urine Drug Screening Among Older Patients With Poor-Prognosis Cancer Near the End of Life. *Journal of Clinical Oncology*. 2023;0(0):JCO.22.01413. doi:10.1200/jco.22.01413
3. Obermeyer Z, Makar M, Abujaber S, Dominici F, Block S, Cutler DM. Association Between the Medicare Hospice Benefit and Health Care Utilization and Costs for Patients With Poor-Prognosis Cancer. *JAMA*. 2014;312(18):1888-1896. doi:10.1001/jama.2014.14950
4. Surveillance E, and End Results Program, National Cancer Insititute. Variable & Recode Definitions. 2023. https://seer.cancer.gov/analysis/
5. Mestdagh F, Steyaert A, Lavand'homme P. Cancer Pain Management: A Narrative Review of Current Concepts, Strategies, and Techniques. *Curr Oncol*. Jul 18 2023;30(7):6838-6858. doi:10.3390/curroncol30070500

© 2025 Rodin R et al. *JAMA Network Open*.
