# Supplementary material for: The 2016 CDC Opioid Guideline and Analgesic Prescribing Patterns in Older Adults With Cancer
Source: JAMA Netw Open. 2025 May 7;8(5):e259043. doi: 10.1001/jamanetworkopen.2025.9043 (PMC12059969; doi:10.1001/jamanetworkopen.2025.9043)
Supplement: Supplement 2. — Data Sharing Statement [file jamanetwopen-e259043-s002.pdf]

Data Sharing Statement

Rodin. The 2016 CDC Opioid Guideline and Analgesic Prescribing Patterns in Older Adults With Cancer. *JAMA Netw Open*. Published May 07, 2025.
doi:10.1001/jamanetworkopen.2025.9043

Data
Data available: No

Additional Information
Explanation for why data not available: The MCBS dataset contains data that have already been collected and re-contacting participants is not possible. Access to the data requires the completion of a DUA, which prohibits any redistribution of the data to other researchers or attempts to re-identify research participants. Researchers may access the data by requesting it and executing and DUA with CMS
